# Innate Immune Responses to Avian Influenza Viruses in Ducks and Chickens

**DOI:** 10.3390/vetsci6010005

**Published:** 2019-01-10

**Authors:** Danyel Evseev, Katharine E. Magor

**Affiliations:** 1 Department of Biological Sciences, University of Alberta, Edmonton, AB T6G 2E9, Canada; evseev@ualberta.ca; 2 Li Ka Shing Institute of Virology, University of Alberta, Edmonton, AB T6G 2E9, Canada

**Keywords:** influenza, duck, tropism, innate immunity, interferon, reservoir host

## Abstract

Mallard ducks are important natural hosts of low pathogenic avian influenza (LPAI) viruses and many strains circulate in this reservoir and cause little harm. Some strains can be transmitted to other hosts, including chickens, and cause respiratory and systemic disease. Rarely, these highly pathogenic avian influenza (HPAI) viruses cause disease in mallards, while chickens are highly susceptible. The long co-evolution of mallard ducks with influenza viruses has undoubtedly fine-tuned many immunological host–pathogen interactions to confer resistance to disease, which are poorly understood. Here, we compare innate responses to different avian influenza viruses in ducks and chickens to reveal differences that point to potential mechanisms of disease resistance. Mallard ducks are permissive to LPAI replication in their intestinal tissues without overtly compromising their fitness. In contrast, the mallard response to HPAI infection reflects an immediate and robust induction of type I interferon and antiviral interferon stimulated genes, highlighting the importance of the RIG-I pathway. Ducks also appear to limit the duration of the response, particularly of pro-inflammatory cytokine expression. Chickens lack RIG-I, and some modulators of the signaling pathway and may be compromised in initiating an early interferon response, allowing more viral replication and consequent damage. We review current knowledge about innate response mediators to influenza infection in mallard ducks compared to chickens to gain insight into protective immune responses, and open questions for future research.

## 1. Introduction

Ducks and waterfowl are the primary hosts of influenza A viruses (IAV) and have a long co-evolutionary history [1,2]. Dabbling ducks are a natural reservoir of low pathogenic avian influenza (LPAI) viruses [2]. IAV are named for their surface proteins, hemagglutinin (HA) and neuraminidase (NA), of which there are now 18 HA and 11 NA antigenically distinct subtypes known (CDC, 2017). The majority of these virus subtypes have been isolated from aquatic birds, including 16 HA and 9 NA subtypes [2,3,4]. Mallard ducks (*Anas platyrhynchos*) have a higher prevalence of LPAI infection than other birds in the wild, harbour these viruses without signs of disease, and shed them into bodies of water [2,5,6,7,8]. The ability to asymptomatically spread influenza A viruses to domestic poultry has earned ducks the moniker “Trojan horses of influenza” [9]. Domestic ducks are often raised in proximity to gallinaceous poultry, and poultry can be extremely susceptible to infection. Among the 16 HA subtypes, only two can cause respiratory and systemic disease in avian hosts, H5 and H7 [10].

By convention, avian influenza viruses are classified as LPAI or highly pathogenic (HPAI) based on their pathogenicity in domestic chickens [11]. LPAI strains with H5 and H7 hemagglutinin subtypes can mutate in gallinaceous poultry to become highly pathogenic to chickens by acquiring a multi-basic cleavage site in the HA, a hallmark virulence factor [12,13]. Influenza hemagglutinin must be cleaved at a specific site by host proteases to make the receptor functional for entry. The amino acid sequence at the HA cleavage site determines how easily it can be cut and by which host proteases. The HAs of LPAI viruses can be cleaved by transmembrane serine proteases and airway trypsin-like proteases, and this limited cleavability restricts infection to certain tissues [14]. The HAs of HPAI viruses with polybasic cleavage sites that contain several consecutive arginine and lysine residues can be activated by a broader repertoire of ubiquitous proteases, such as furin and plasmin, and can thus infect a wider range of tissues and produce more serious diseases [15,16,17]. In chickens or quail, the H5 or H7 hemagglutinin subtypes may occasionally acquire mutations that make them more similar to those of human seasonal viruses, making the virus more likely to zoonotically infect humans [18,19].

Mallards are more resistant to disease caused by influenza than most other species, including those caused by highly pathogenic avian influenza viruses [20,21,22]. However, even mallard ducks sometimes succumb to fatal infection by certain strains of H5 HPAI viruses, particularly certain strains belonging to the Eurasian lineage of HPAI H5 viruses tracing back to the Chinese strain A/goose/Guangdong/1/1996 [12,23,24,25]. Poultry outbreaks and human infections with HPAI H5N1 strains of this lineage began occurring in large numbers in Southeast Asia between 2003 and 2004. Since then, the viruses have been spread to Africa, Europe, and North America, and have become endemic in poultry in China, Indonesia, Vietnam, India, Bangladesh, and Egypt [26]. More recently, in 2014, a reassortant H5N8 that could kill domestic ducks caused several poultry outbreaks in South Korea [27,28], spread to western Europe, and arrived on the west coast of North America with migratory waterfowl later that same year [29]. It is worth noting, however, that the spread of highly pathogenic influenza strains results from both wild waterfowl migration and human movement of infected poultry [30], and that the pathogenic strains do not appear to persist for long in wild birds in nature [9].

The co-evolution of mallard ducks with influenza viruses has produced unique host–pathogen interactions that allow ducks to act as asymptomatic carriers of LPAI viruses, and to often survive HPAI virus infections. In this review, we discuss the importance of tissue tropism differences and protective innate immune responses in the early part of influenza infection toward susceptibility and survival. We compare these aspects between ducks and the extremely susceptible chickens to highlight potential mechanisms of disease resistance and suggest some intriguing questions for future research.

## 2. Sialic Acid Receptor Distribution and Influenza Virus Tissue Tropism in Ducks and Chickens

Tissue tropism is partly determined by the distribution and availability of glycoprotein receptors with terminal sialic acid in appropriate linkages, which influenza hemagglutinin molecules bind to in order to gain entry into cells. Sialic acid α-2,3-galactose is preferred by avian strains and sialic acid α-2,6-galactose is preferred by human-adapted influenza viruses. An exhaustive study by Costa and colleagues examined the tissue distributions of both α-2,3-Gal SA receptors and α-2,6-Gal SA receptors in seven bird species [31]. The authors used lectins to investigate the SA receptor distributions in the nasal cavity, tracheal, and lung tissues, as well as in the duodenal, ileal, cecal, and colonic tissues of chickens, common quail, red-legged partridges, turkeys, golden pheasants, ostriches, and mallard ducks. Both α-2,3-Gal and α-2,6-Gal SA receptors were present in the respiratory and intestinal tissues of all the galliformes, whereas α-2,6-Gal SA receptors were absent from the intestine of mallards and from virtually all ostrich tissues. Consequently, human H1N1 virions did not bind to duck intestinal tissues, while chickens, pheasants, and quail in particular had the highest virion adherence in all tested tissues. Kuchipudi et al. [32], using similar methods, found both types of sialic acid receptors in many tissues of chickens and ducks, but in chicken tracheas α-2,6-Gal was the dominant receptor type, while in ducks the α-2,3-Gal receptors were most abundant. These results suggest that chickens are more important hosts for the generation of influenza viruses with increased ability to infect humans.

Low-pathogenicity avian influenza viruses replicate primarily in the intestinal tract of ducks [33], where they do not appear to cause lesions, and are circulated by the fecal–oral route in the ducks’ aquatic environment [6,34,35,36,37]. Although the preferred α-2,3-Gal sialic acid receptors for LPAIV hemagglutinin are abundant in both the airways and the lower intestine, both naturally and experimentally infected ducks display virus replication principally in the lower gut and long-term shedding from the cloaca only [31,35,37]. Experiments with LPAIV subtypes H3N8, H5N2, H2N3, and H13N6 all show that the airways do get infected through the mucosa of the head, as occurs in natural dabbling behaviour, and mild tracheitis and pneumonia are detectable in the first two days after infection, but all the viruses replicate for longer and to higher titres in the colon, cecum, and bursa of Fabricius [36,38], while the virus becomes undetectable in tracheal swabs after 3 or 4 days [36,37,38]. In experimentally infected wild birds of different species, mallard-origin LPAI viruses were shed primarily from the gastrointestinal tract of mallards, but often found in oropharangeal swabs of wood ducks, redheads and laughing gulls [39]. When chickens and ducks were infected with LPAI A/Mallard/British Columbia/500/2005 (H5N2), in both species the virus replicated to similarly high titres in the intestines, but the titre in duck lungs was of 2–3 orders of magnitude lower than the titre in chicken lungs [40]. Cornelissen et al. infected 3-week-old chickens and ducks by the intratracheal route with LPAI A/chicken/Italy/1067/99 (H7N1) and similarly found significantly higher viral RNA quantities in the lungs of chickens versus ducks, but higher viral RNA quantities in the gut and bursa of ducks [41]. Low pathogenic viruses preferentially replicate in the intestinal tract of mallard ducks but replicate to high titre in both the lungs and intestines of chickens and other birds.

Highly pathogenic avian influenza viruses replicate primarily in the airways of anseriformes and have a higher propensity to spread to other tissues and organs systemically, as in other animals [37,42,43,44,45,46]. However, the systemic spread and damage by highly pathogenic influenza viruses in ducks is more often limited than in other species. A study of naturally infected chickens, quail, and ducks, by HPAI H5N1 in Thailand in 2004, found that viral antigens were often distributed throughout the tissues of the chickens and quail, and less often in all the duck tissues except skeletal muscle and brain tissue [42]. HPAI viruses display weaker endothelial tropism in ducks than in galliformes. In 2014, Short and colleagues reviewed histological data from experimental and natural infections and noted a striking difference in HPAIV endothelial cell tropism between galliform and anseriform birds [13]. In galliformes, HPAI viruses readily infect respiratory endothelium and the capillary beds of many organs, whereas in ducks HPAI viruses rarely infect endothelial cells. The authors suggest that endothelium destruction in galliform birds contributes to increased hemorrhage and edema observed in those species. Mallards also appear able to tolerate a certain amount of systemic HPAI replication, particularly strikingly in the brain and central nervous system (CNS), and still successfully clear the virus [43]. Vidaña and colleagues infected Muscovy ducks, European quail, and chickens with A/Anhui/1/1203 (H7N9), an avian influenza virus that was highly pathogenic in humans [47]. All of the birds shed more virus from the airways than from the cloaca, and none developed symptoms or died. However, the quail had more severe microscopic lesions and more viral antigen in the upper airways, which the authors correlated to a higher density of α-2,6-Gal SA receptors. Interestingly, the ducks in this study successfully transmitted the virus to contact animals, but the chickens did not, despite shedding more virus.

## 3. Factors Affecting Susceptibility to Disease in Mallards

### 3.1. Susceptibility of Different Anseriform Species

The extent of systemic spread and replication determines disease severity, and can depend on the viral strain, anseriform species, and age [46,48]. Striking differences in susceptibility have been observed between different anseriform species in both infected wild birds and experimentally challenged birds. In a comparison of mute swans, greylag geese, ruddy shelducks, mandarin ducks, and mallard ducks infected with HPAI A/chicken/Korea/IS/06 (H5N1), Kwon and colleagues [48] found that all the swans and shelducks died and had lesions and viral antigen present in multiple organs. All of the mallard ducks and two out of three mandarin ducks survived while shedding the virus from the tracheas and cloacas, but with no histological lesions or viral antigen detectable in the tissues. The dead mandarin duck had viral antigens in the brain and pancreas, and lesions in multiple organs including in the CNS and the heart. Interestingly, the greylag geese in the study all developed neurological symptoms and had viral antigen and lesions in the CNS, but in no other tissues, and all survived the duration of the experiment. Ducks of the genus *Anas*, geese of the genus *Anser*, and swans of the genus *Cygnus* all have relatively similar distributions of both α-2,3-Gal and α-2,6-Gal SA receptors in their respiratory and digestive tracts, which do not appear to account for the differing susceptibilities of these species [49]. Muscovy ducks (*Cairina moschata*) are also more susceptible than mallard ducks to HPAI virus disease [50,51]. Differences in susceptibility between different duck species are not easily explained by receptor distributions or innate responses, but the mallard duck is more resistant to influenza viruses than other Anseriformes.

### 3.2. Age of Birds

The age of the birds is a significant factor in their susceptibility, with younger birds being more susceptible. 2-week-old Pekin ducks had a higher mortality rate than 5-week-old Pekin ducks from infection with two HPAI H5N1 viruses (A/Egret/HK/757.2/2002 and A/Thailand PB/6231/2004), and the surviving older ducks had fewer histological lesions and less systemic spread of the viruses in both cases [46].

### 3.3. Viral Strain-Dependent Differences in Infection Outcome

Different HPAI virus strains produce different outcomes in mallard duck hosts. A comparison of the lethality of the viruses from the dominant clades of H5N1 viruses in Pekin ducks showed that subclade 2.3.4 viruses were the most lethal [52]. All viruses tested in this study could be lethal to ducks at high inoculation doses, but A/Duck/Laos/25/2006 (H5N1 clade 2.3.4) killed all ducks within 7 days, and was lethal even at low doses. Bingham et al. [43] infected Pekin ducks with two strains of HPAI H5N1 from the Eurasian lineage—A/Muscovy duck/Vietnam/453/2004 and A/duck/Indramayu/BBVW/109/2006—and found that the Vietnamese strain caused more severe disease, longer fever, and had much greater antigen accumulation in skeletal and heart muscle, organs, and the CNS. Similar to the non-fatal CNS involvement in greylag geese [48], Bingham et al. [43] noted in their study that the Indonesian (BBVW/109) strain caused encephalitis in ducks, but did not produce any overt disease symptoms beyond transient fever.

In summary, low pathogenicity avian influenza viruses replicate preferentially in the intestinal tracts of ducks and cause no apparent tissue damage or disease signs. Highly pathogenic viruses replicate primarily in the airways of ducks, and can spread systemically to organs, skeletal muscle, and the central nervous system. Mallard ducks are more resistant than other bird species to HPAI virus disease, and disease severity depends on host age and fitness and the viral strain. Interestingly, members of *Anatidae* appear to tolerate a certain amount of influenza-induced encephalitis, and HPAI viruses do not appear to replicate in duck endothelial cells, as they do in those of chickens and mice.

## 4. Innate Immune Signaling—Pro-Inflammatory and Interferon Responses

**Figure 1:**
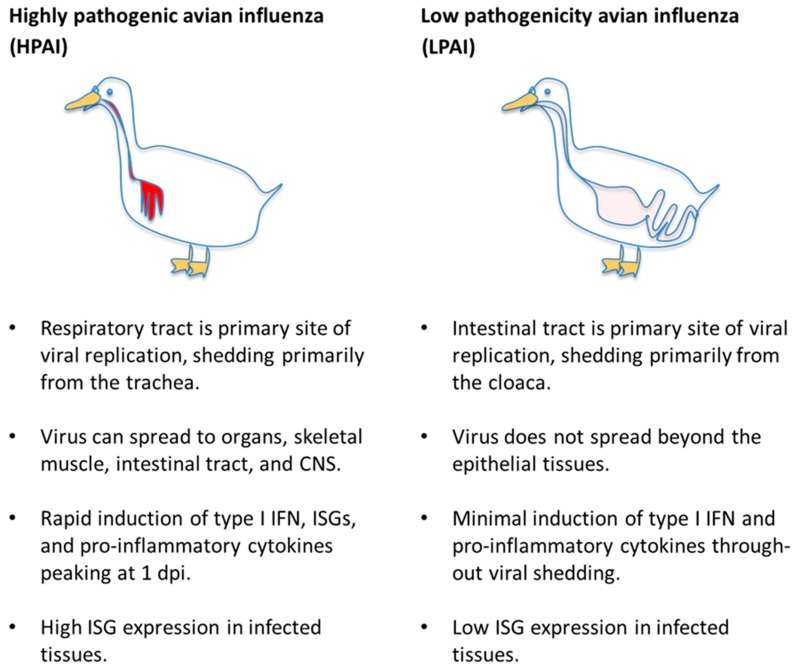
Summary of the generalized innate immune responses of ducks to highly pathogenic and low pathogenicity avian influenza viruses. ISG: interferon-stimulated genes.

Since death from highly pathogenic influenza infection is a matter of days in susceptible birds and mammals, too early for adaptive immune responses to account for the survival advantage of immunologically-naïve ducks, it is worthwhile to compare the innate immune signaling in that early period. Innate immune responses to highly pathogenic influenza viruses in general can be divided into two modalities—protective type I and type III interferon signaling that induces an anti-viral state and controls inflammation or an aberrant hyper-activation of pro-inflammatory signaling that likely contributes to pathology. HPAI virus-infected ducks appear less prone than other species to hyper-inflammation and its associated pathology and produce an early and apparently protective type I interferon response. LPAI virus infections cause virtually no pathology in ducks, and accordingly do not induce strong innate immune responses. The generalized innate immune responses of ducks to HPAI and LPAI viruses are summarized in Figure 1.

### 4.1. Inflammation

Acute inflammation is a broad response to cellular damage or infection in which physical and chemical signals bring an influx of immune cells and fluid from the circulatory system to the site of injury [53]. Many pro-inflammatory cytokines, like IL-6 and TNF-α, are released by stressed cells to induce leakiness in the capillaries around the injury and to act as chemoattractants and stimulators to macrophages, neutrophils, and other leukocytes, which can then combat pathogens and clear out debris. However, the excessive infiltration of cells and fluid into the submucosal tissues of the airways can compromise oxygen exchange and produce hypoxia and respiratory distress syndrome, which have been implicated in severe human H5N1 infections [54,55,56]. Uncontrolled inflammation in other sensitive tissues, like in the CNS, can be equally dangerous [57]. Since inflammation is a complex and multifactorial process, many influenza virus studies look at the induction of pro-inflammatory cytokines specifically as a proxy for inflammation in general. An excessive inflammatory immune response, “cytokine storm,” is suspected to be a major disease mechanism in fulminant HPAI virus infections in humans [58,59,60], mice [61,62,63,64], and birds, including ducks [65,66].

Ducks are susceptible to certain HPAI virus strains in the modern Eurasian lineage and may also manifest symptoms of inflammatory pathology. In a comparison of non-vaccinated Pekin and Muscovy ducks infected with HPAI A/Dk/Nam Dinh(VietNam)/NCVD-88/2007 (H5N1), Cagle et al. saw faster mean death times and more severe symptoms in Muscovy ducks, which also produced more IL-6 mRNA in their spleens [50]. However, the authors noted that the infected Pekin ducks, who also all died within several days, had higher body temperatures than the Muscovy ducks, and higher nitric oxide concentrations in the blood at 2 dpi. Nitric oxide synthesis is stimulated by interferon gamma (IFN-γ) and TNFα signaling [67]. In a follow up experiment with Pekin ducks, Muscovy ducks, and one wild mallard infected with A/duck/NauGiang/NCVD07-12/2007 (H5N1), Cagle et al. again saw a similar trend where the Muscovy ducks died two days earlier than the others, and had slightly higher IL-6 mRNA upregulation in the spleen [51]. Mortality rates were high in mallard ducks infected experimentally with the A/duck/D4AT/Thailand/71.1/2004 (H5N1) and A/Vietnam/1203/2004 (H5N1) viruses [68]. In a sub-lethal challenge with these viruses, our quantitative PCR analysis of the infected spleens and lungs showed only a transient upregulation of *IL1B* and *IL6* mRNA on the first day post-infection, which returned to control levels by the second or third day [69]. Low-pathogenic influenza viruses do not induce pro-inflammatory cytokines in duck tissues, primary duck lung cells, or peripheral blood mononuclear cells [41,70,71].

The degree of inflammation in ducks depends on the viral strain. Muscovy ducks infected with different Eurasian lineage HPAI H5N1 viruses had very different fates [62,66]. Ducks infected with A/Dk/Guangdong/383/2008 (HA clade 2.3.2, genotype 11) suffered 60% mortality and neurological symptoms while ducks infected with A/duck/Guangdong/212/2004 (HA clade 9, genotype 3) did not die and displayed fewer symptoms [62]. Both viruses replicated systemically, in the brains, spleens, kidneys, and pancreases of the Muscovy ducks, but Guangdong383 replicated to higher titres and induced much higher gene expression levels of IL-6 and IL-8 [66]. Thus, IL6 gene expression correlates with severity of the infection in ducks.

Ducks show modest upregulation of cytokine gene expression to many highly pathogenic strains. There is accumulating evidence that ducks generally control inflammation better than chickens in response to highly pathogenic influenza infection. Using HPAI A/turkey/Italy/4580/99 (H7N1) to infect chickens and ducks, Cornelissen et al. observed lethal systemic infections in the chickens and asymptomatic infections in the ducks [72]. In the chickens, along with higher viral loads in lung, brain and spleen tissues, the authors saw high pro-inflammatory cytokine upregulation at 2 and 3 days post-infection (dpi). In the ducks, there was a moderate and brief upregulation of cytokines within the first dpi, similar to our observations [69]. In side-by-side infections of chickens and ducks with two Eurasian lineage HPAI H5N1 viruses, IL-6 and other pro-inflammatory cytokines were more potently induced in chickens and correlated with higher mortality [65]. A similar trend was observed in vitro, in chicken and duck embryonic fibroblasts or primary lung cells infected with H5N1 [71,73]. Chicken primary skeletal muscle myotubes produce more IL-6 and IL-8 than duck primary myotubes, when infected with either HPAI H5N1 or LPAI H2N3 viruses [74]. Burggraaf and colleagues compared nitric oxide production and iNOS gene expression in chickens and ducks infected with A/Muscovy duck/Vietnam/453/2004 (H5N1) [75]. They found higher NO concentrations in chicken serum, and significantly higher iNOS mRNA upregulation in most tissues of chickens compared to ducks. The observation that HPAI influenza viruses do not display a strong endothelial tropism in ducks [13] may also be relevant for the control of inflammatory signaling.

In summary, high levels of pro-inflammatory cytokines seem to correlate with symptom severity in ducks, as well as in other species, but mallards seem less prone than other birds to hyper-inflammatory responses to highly pathogenic avian influenza viruses. Low-pathogenic strains elicit virtually no pro-inflammatory signaling in ducks and chickens.

### 4.2. Type I and III Interferon Responses

Type I interferons (IFN-α and IFN-β) are produced when influenza virus RNA is detected by host cell pattern recognition receptors [76]. An interferon signal from epithelial cells at the initial site of infection acts in a paracrine fashion to induce an anti-viral state in the surrounding cells. It does this by stimulating the expression of many multifunctional interferon-stimulated genes (ISGs) that modulate host cell metabolism and interact with viral components directly to suppress replication [77,78,79,80,81,82]. This is an important first checkpoint to delay viral replication and spread while other components of immunity become activated. There is a lot of evidence in humans and mice that an early type I interferon response is crucial for controlling the influenza virus [76,83,84,85,86,87]. There is also a subset of leukocytes called plasmacytoid dendritic cells (PDCs) that can secrete large amounts of IFN-α into the circulatory system, and are considered “professional” type I interferon producers [88,89]. IFN-α secretion in PDCs is triggered by toll-like receptor 7 (TLR7). However, some evidence suggests that these circulating cells do not necessarily act as sentinels of infection but are activated secondarily and serve to amplify the immune response and modulate T-cell activity [90].

Type I interferon is also known to oppose pro-inflammatory signaling [91,92]. The relationship between type I interferon and inflammation is complex and context-dependent, as reviewed by Trinchieri [93]. However, in the context of influenza infection, type I interferon signaling seems to promote the secretion of the anti-inflammatory cytokine IL-10 [94] and to oppose neutrophil recruitment [95].

Ducks typically manifest a rapid upregulation of type I interferon in response to HPAI infection, which peaks on the first day. Pekin ducks infected with highly pathogenic Eurasian lineage H5N1 viruses upregulated *IFNA* and *IFNB* genes early, by 1 dpi, in lungs and spleens, by qPCR [37,51,69]. Similar to their pro-inflammatory responses, the interferon spikes came down by the second and third days of infection, but a sustained induction of key ISGs remained [69]. A transcriptome analysis of H5N1-infected ducks also showed a large and early interferon response underway in the lungs at 1 dpi and the induction of ISGs persistent through 3 dpi [40]. The ducks also produced interferon in their ilea, though not as rapidly as in their lungs, and ISG induction in both tissues was higher than in similarly infected chickens. The transcriptome comparison showed that the first responses to influenza in chickens and ducks are dramatically different—the genes expressed in the chickens were mostly related to T-cell and B-cell activation, whereas the early genes expressed in ducks were all related to pathogen-associated molecular pattern recognition and interferon signaling. Direct comparison is complicated by the need to challenge highly susceptible chickens with a much lower infecting dose than ducks. Kumar et al. [96] infected domestic ducks with two highly pathogenic Eurasian lineage IAV strains that differed in the severity of disease that they caused. A/duck/Tripura/103597/2008 (H5N1) is a HPAI strain that belongs to clade 2.2.2.1 [97,98] and possesses a polybasic HA cleavage site (residues RRRKKR at positions 341–346, GenBank accession: GU252822). However, it caused milder disease in experimentally infected ducks compared to A/duck/India/02CA10/2011 (H5N1) (clade 2.3.2). The authors found upregulation of important antiviral interferon-stimulated genes in the lungs of Tripura/103597-infected ducks, but not in India/02CA10-infected ducks at 5 dpi, showing that ISG expression correlates inversely with disease severity in ducks [96]. Muscovy ducks, which are more susceptible to influenza pathology, have higher pro-inflammatory responses to HPAI viruses compared to Pekin and wild mallard ducks, and also have lower IFN-α responses in their spleens [51]. Recombinant duck IFN-α was recently shown to inhibit influenza virus replication in duck fibroblasts in vitro, and to stimulate the expression of ISGs in the brains and spleens of treated ducks in vivo [99]. In ducks that survive HPAI infection, an early type I interferon response is observed, which probably slows down viral spread and limits tissue damage, and therefore inflammation.

The single known type III interferon, IFN-λ, shares common upstream regulatory elements with type I interferons, is secreted by many of the same cell types, and induces many of the same ISGs, but signals through a different receptor on target cells primarily in mucosal linings [100,101]. Interferon lambda was upregulated early in HPAI H5N1-infected duck lungs and spleens [69], and was upregulated 20-fold in the lungs of Chinese geese infected with LPAI H9N2 [102].

LPAIV are typically weak inducers of interferon in ducks [37,40]. Cornelissen and colleagues actually found earlier and more robust IFN-α mRNA induction in chicken lungs, guts, and bursas than in those of ducks, infected with LPAI A/chicken/Italy/1067/1999 (H7N1) [41]. Low-pathogenic influenza A/turkey/Italy/977/1999 (H7N1) replicated in duck embryonic fibroblasts and suppressed interferon, whereas a recombinant version expressing a truncated viral non-structural protein 1 (NS1) induced interferon and replicated poorly [103]. In another infection study using the same LPAI A/turkey/Italy/977/1999 (H7N1) virus, there was little IFN-α produced by hematopoietic cells (potential counterparts of PDCs or professional interferon-producing cells) in duck intestinal tissues [104]. IFN-α-producing cells could barely be detected by in-situ hybridization in the mucosal tissues of the wild-type virus-infected ducks but could be detected in mucosa following oral treatment with a TLR7 agonist. In summary, low pathogenicity influenza viruses do not induce significant interferon or pro-inflammatory signaling in the intestinal tract of ducks, where they replicate.

Chickens also mount robust IFN-α responses to HPAI H5N1 infection, within 1 to 2 dpi, but fail to control viral replication and succumb to an apparent cytokine storm [105,106,107]. The key difference between chicken and duck early type I interferon induction, which we propose contributes to this striking difference in outcome, is related to their pattern recognition receptors as discussed in the next section. LPAI infections in chickens, as in ducks, induce lower interferon responses than HPAI infections, in a strain-dependent manner [106,108].

## 5. Influenza Virus Pattern Recognition—RIG-I-Like Receptors

RIG-I like receptors (RLRs) are a family of three cytoplasmic RNA sensing proteins: LGP2, MDA5 and RIG-I. All three members share a DExD/H box RNA helicase domain that recognizes dsRNA species and a C-terminal repressor or regulatory domain (RD) that undergoes a conformational change when the helicase domain binds to its target. MDA5 and RIG-I, but not LGP2, possess a tandem pair of N-terminal caspase activation and recruitment domains (CARD) that initiate a signaling pathway leading to type I interferon expression, once released from RD repression. In a positive feedback loop, all three RLRs are interferon-inducible in ducks [109,50], in fish [110], and in humans [111,112,113,114,115,116].

### 5.1. RIG-I

RIG-I is the most important of the RLRs for recognizing influenza viruses and is responsible for the earliest epithelial type I interferon responses in an influenza infection [117,118,119,120]. RIG-I recognizes 5′-triphosphorylated RNA “panhandle” structures formed by the complementary ends of each influenza genome segment [121,122]. Short aberrant RNAs (mini viral RNAs) produced by influenza polymerases due to any dysregulation or erroneous activity in the replication process act as strong agonists of RIG-I [123]. The same is true of RNA species in defective interfering (DI) particles [124,125], which are virions containing incomplete or faulty genomes that also arise from replication or packaging errors [126]. Recently, a subpopulation of RIG-I that resides in the nucleus during influenza infection and elicits an interferon response from that compartment has been identified in human cells [127]. Finally, independent of its IFN signaling function, cytoplasmic RIG-I (but not MDA5) can act as an antiviral effector protein by binding to incoming IAV nucleoprotein and delaying the first cycle of replication [128].

In ducks, RIG-I is ubiquitously expressed, especially in mucosal tissues, and the highest constitutive expression in healthy Muscovy ducks is in the trachea [129]. Induced by interferon, RIG-I mRNA is highly upregulated in Pekin duck lungs early in HPAI H5N1 infections [109,69]. Transcriptome analysis and qPCR indicate that RIG-I signaling pathway genes play a prominent role in mallard responses to HPAI H5N1 viruses [40,130]. In Muscovy ducks, RIG-I expression increased in brains, spleens, lungs, and bursae in response to infection with LPAI A/Chicken/Shanghai/010/2008 (H9N2) [129]. In contrast, RIG-I expression was not significantly induced in Pekin duck intestines in response to LPAI infection [109].

Chickens lack the RIG-I gene, and this almost certainly contributes to their extreme susceptibility to influenza disease [109,131]. When chickens and ducks were infected with LPAI H7N1, the chickens highly upregulated MDA5 and TLR7 in their lungs and yet produced higher viral titres, while the ducks upregulated RIG-I and restricted viral replication in their lungs [41]. Highlighting the importance of RIG-I, a human patient who had a severe infection during the 2009 H1N1 pandemic was found to have a signaling and RNA-binding-deficient variant of RIG-I [83]. Correlating with increased pathology, cultured peripheral blood mononuclear cells and macrophages from that patient showed an elevated IL-6 and TNF-α response to PR8 infection, compared to controls. Kallfass and colleagues showed in mice that epithelial cells are the primary source of IFN-β at the earliest timepoints of influenza infection, if they are not successfully inhibited by viral NS1, which targets the RIG-I pathway [132]. Thus, chickens, without RIG-I, are predicted to be deficient in IFN-β at the first critical epithelial cell barrier. This inference is supported by the fact that chicken splenocytes (likely plasmacytoid dendritic cells and other specialized immune cells) respond with IFN-α to HPAI A/chicken/Yamaguchi/7/2004 (H5N1) in vitro, while chicken fibroblasts do not [106]. It seems clear from the fates of HPAI-infected chickens that IFN-α signaling by specialized myeloid cells is not sufficient on its own to limit viral replication in the first cycles of infection.

### 5.2. MDA5

MDA5, the second member of the RLR family, detects uncapped mRNA and long dsRNA (>1 kb in length) in the cytoplasm [133,134]. Murine and Muscovy duck MDA5 receptors demonstrably contribute to influenza virus suppression [135,136]. Muscovy duck MDA5 is ubiquitously expressed in tissues and is highly induced in the lungs on the first day of HPAI H5N1 infection [136]. The CARD domains of duck MDA5 on their own are constitutively active and induce IFN-β and ISG production in duck embryonic fibroblasts (DEFs) in vitro. Chickens upregulate MDA5 expression in response to both HPAI and LPAI infections [41,72], but chicken fibroblasts produce little IFN-β when infected and produce more when reconstituted with recombinant duck RIG-I [109,106]. Furthermore, siRNA knockdown of MDA5 in chicken DF-1 cells did not impact influenza virus replication [137], suggesting that MDA5 may only partially compensate for the lack of RIG-I signaling in chickens.

### 5.3. LGP2

The third member of the RLR family, LGP2, may act as a positive or negative regulator of RIG-I. It lacks its own CARD domains but can oppose RIG-I signaling either by competing for RNA ligands or by directly repressing its CARD domains with its own regulatory domain [138,139,140,141,142]. On the other hand, there is also evidence for context-specific positive regulatory roles of LGP2 in the RIG-I/MDA5 pathways [143,144]. Muscovy duck and mallard duck LGP2 proteins share 88% amino acid sequence identity, but both are only 30% similar to RIG-I at the C-terminal regulatory domain. In resting Muscovy ducks, LGP2 mRNA is highly transcribed in the intestines, crop, and caecum, and is restricted in the brain and heart [145]. Expression is highly induced in the brain, spleen, and lungs on the first two days post infection with HPAI A/Duck/Guangdong/212/2004 (H5N1), but the functional consequences have yet to be determined.

### 5.4. RLR-Signaling Pathway

**Figure 2:**
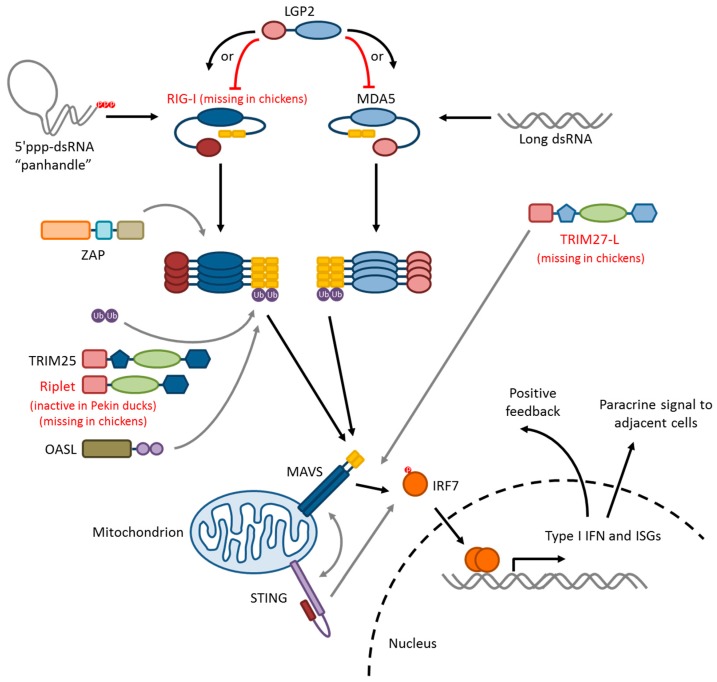
Duck RIG-I-like receptor (RLR) family signaling pathway. The duck RLR family consists of three cytoplasmic receptors: RIG-I, MDA5, and LGP2. RIG-I and MDA5 detect viral RNA species and initiate interferon signaling. LGP2 does not possess a signaling domain and may act as a positive or negative regulator of the other two. RIG-I recognizes 5′-triphosphorylated RNA “panhandle” structures formed by the complementary ends of each influenza genome segment. MDA5 recognizes long double-stranded RNA. Ligand recognition leads to oligomerization of the receptors and releases the N-terminal caspase activation and recruitment domains (CARD) domains (shown as yellow rectangles) from repression by the C-terminal regulatory domain. Cytoplasmic zinc-finger antiviral protein (ZAP) protein may interact directly with RIG-I to stabilize RNA-bound tetramers. Short K-63-linked polyubiquitin chains synthesized by tripartite motif-containing protein 25 (TRIM25) stabilize the RIG-I CARD domain oligomers, which interact with similar CARD domains on mitochondrial MAVS. Duck Riplet appears to be missing its catalytic domain and likely cannot synthesize polyubiquitin chains. 2′-5′-oligoadenylate synthase-like protein (OASL) may stabilize RIG-I CARD domain oligomers with its own C-terminal ubiquitin-like domains in the absence of TRIM25-mediated ubiquitination. Activation of MAVS proteins leads them to multimerize in turn, and to serve as an assembly platform for a kinase cascade, which terminates in the phosphorylation of the transcription factor IRF7. A unique duck TRIM gene, TRIM27-L, promotes RIG-I signaling through an undefined interaction downstream of MAVS. Phosphorylated IRF7 dimerizes and is translocated to the nucleus where it initiates transcription of type I and type III interferon and interferon-stimulated genes. The expressed interferon is secreted to induce an antiviral state in the neighbouring cells and can also amplify RLR signaling in a positive feedback loop, since the pathway components are themselves interferon-inducible. RIG-I signaling activates stimulator of interferon gene (STING), an adaptor protein involved in sensing DNA viruses, which cross-talks with the RIG-I pathway. STING activation, in turn, prolongs the type I IFN response.

The duck RLR signaling pathway is summarized in Figure 2. RIG-I and MDA5 both signal via a homotypic CARD–CARD interaction with the CARD domain of a signaling adaptor, mitochondrial antiviral-signaling protein (MAVS), which leads to the activation and nuclear translocation of interferon regulatory factors IRF3 and/or IRF7. The mechanism of signal initiation involves the tandem CARDs of four activated RIG-I or MDA5 proteins nucleating a helical assembly of MAVS CARDs into filamentous structures, a process called “molecular imprinting,” in both ducks and humans [146]. This is a spontaneous process, as demonstrated by the constitutive activity of isolated RIG-I or MAVS CARD domains that are free from auto-repression [147,136]. MAVS oligomers then serve as the assembly platform for downstream signaling intermediates. The interacting surfaces between RIG-I-2CARD:MAVS–CARD and MAVS–CARD:MAVS–CARD are not highly conserved between ducks and humans, but T175K/T176E mutations in duck RIG-I permit signaling through human MAVS in HEK293T cells [146]. Furthermore, wild-type duck RIG-I–2CARD can interact with chicken MAVS in the chicken DF-1 cell line, allowing the RIG-I signaling to be recapitulated [109,129,147,148,149]. Full-length mallard RIG-I transfected into DF-1 cells restores detection of 5′ppp RNA and results in lower titres of LPAI A/Mallard/British Columbia/500/2005 (H5N2) and HPAI A/Vietnam/1203/2004 (H5N1) compared to infected controls [109]. Induction of IFN-β and ISGs is higher in RIG-I-transfected DF-1 cells, by qPCR, microarray, and transcriptome analyses [148,149]. Muscovy duck RIG-I is 97.4% identical to mallard RIG-I at the amino acid level, and also induces IFN-β signaling, and resistance to LPAI strain A/Chicken/Shanghai/010/2008 (H9N2) in transfected DF-1 cells [129]. IRF3 is apparently missing in birds [131], and so avian MAVS is likely signaling through IRF7. Indeed, there is increased nuclear localization of chicken IRF7-mCherry in DF-1 cells upon stimulation of MAVS signaling [150]. Thus, RLR signaling in ducks mostly resembles the human paradigm.

The RLRs are an important family of pattern recognition receptors for early epithelial antiviral responses. In particular, duck RIG-I is associated with anti-influenza innate immunity, and the lack of RIG-I in chickens may have far-reaching immunological consequences.

## 6. Modifiers of RIG-I/MAVS Signaling

Here we discuss modifiers of RIG-I signaling in ducks and chickens. Information about the chicken orthologues is given, where available, from an evolutionary interest, keeping in mind that RIG-I is absent from the chicken genome.

### 6.1. Ubiquitin Ligases: TRIM25, Riplet, and RNF125

RIG-I signaling is regulated by ubiquitination. Three ubiquitin ligases—tripartite motif-containing protein 25 (TRIM25), RING finger protein 135 (RNF135 or Riplet) and RING finger protein 125 (RNF125)—regulate RIG-I [151,152,153,154,155]. TRIM25 and Riplet are positive regulators, stabilizing activated RIG-I by the addition of K63-linked ubiquitin chains [152,153,154]. RNF125 mediates K48-linked polyubiquitination, which leads to the degradation of RIG-I [151]. To our knowledge, no studies of duck or chicken RNF125 have been published.

TRIM25 stabilizes the RIG-I–2CARD: MAVS–CARD helical structure by furnishing short chains of K63-linked ubiquitin molecules that bind along the outside of the structure [152,146]. Structural studies have shown that these short ubiquitin chains do not necessarily have to be covalently anchored to RIG-I–CARD domains in order to interact and stabilize the assembly [156,157]. A new function of TRIM25 has recently been described in humans—a nuclear subpopulation of human TRIM25 was identified, which bound directly to influenza nucleoprotein and physically prevented RNA strand elongation by the viral polymerase [158].

In ducks, TRIM25 ubiquitinates RIG-I–2CARD domains and amplifies MAVS signaling [147]. Two amino acids in the 2CARD—K167 and K193—covalently anchor K63-linked ubiquitin chains, but mutation of both of them does not abolish signal amplification as long as interaction with TRIM25 is conserved. Thus, duck TRIM25 provides both anchored and unanchored K63-linked polyubiquitin chains to potentiate RIG-I signaling. Direct viral restriction by nuclear TRIM25 has yet to be demonstrated in ducks. Rajsbaum et al. [155] demonstrated that chicken TRIM25 contributes to IFN-β production in chicken cells during influenza virus infection in vitro, but did not identify a mechanism, which remains an open question. In human cells, Gack et al. [152] showed that only RIG-I, and not MDA5, is ubiquitinated by TRIM25, but perhaps the situation is different in chickens.

Riplet stabilizes RIG-I activation differently from TRIM25, by ubiquitinating the RIG-I C-terminal repressor domain [153], and is necessary for maximal type I interferon induction in response to influenza virus infection in mice. [154,155]. Riplet is missing from the chicken genome, and appears to lack exon 1 in Pekin ducks [131]. Exon 1 encodes the catalytic RING domain, meaning that Pekin duck Riplet would presumably be unable to transfer ubiquitin [154].

In summary, TRIM25 is functional and contributes to type I interferon responses in ducks and chickens, though it is still unclear how in the latter. Riplet appears to be missing in chickens and may be non-functional in ducks, and nothing is yet known about RNF125 in either species.

### 6.2. TRIM27-L and TRIM27.1

A pair of TRIM27-like genes that modulate the RIG-I-mediated interferon response were identified in ducks, one of which is absent in chickens and turkeys [159]. TRIM27-L, unique to ducks, strongly activates RIG-I–2CARD signaling in DF-1 cells, while TRIM27.1, which is shared with chickens and turkeys, weakly opposes signaling. In a co-transfection experiment, TRIM27-L activation overcame TRIM27.1 inhibition.

### 6.3. ZAP

Zinc-finger antiviral protein (ZAP or ZC3HAV1) is a direct viral restrictor and a positive regulator of RIG-I signaling. In humans, ZAP directly binds human immunodeficiency virus type 1 (HIV-1) mRNA and targets it for degradation [160,161]. ZAP also potentiates human RIG-I signaling by interacting directly with the RIG-I helicase and repressor domains to stabilize RNA-bound oligomers [162]. Chicken ZAP is structurally homologous to mammalian orthologues, and inducible by IFN-α or poly(I:C) in chicken splenocytes [163]. ZAP mRNA was upregulated in chicken lungs and spleens in response to infection with HPAI A/Muscovy duck/Vietnam/453/2004 (H5N1) [163]. To our knowledge, no investigation of duck ZAP has been published to date.

### 6.4. STING

Stimulator of interferon gene (STING) is an adaptor molecule that links intracellular pathogen RNA and DNA detection to NF-κB and IRF activation, and IFN-β production [164,165,166]. Similarly, to mitochondrial MAVS in the RLR pathway, STING resides on the endoplasmic reticulum and mitochondrial outer membrane and activates IRF3/7 in response to foreign nucleic acid detection [164,166,167]. STING directly detects cyclic dinucleotides, which can be of bacterial origin or generated as a secondary signal in response to foreign DNA detection by cyclic GMP–AMP synthase (cGAS) [168,169,170]. Activated STING moves to perinuclear vesicles to initiate a phosphorylation cascade that leads to the nuclear translocation of IRFs [165]. There is significant cross-talk between mammalian RIG-I and STING signaling through several indirect pathways and through a direct interaction between STING and MAVS [167]. In human and mouse cells, RIG-I signaling increases STING expression, which in turn prolongs IFN-β production [171]. Chicken STING overexpression in DF-1 cells activates IRF7 and inhibits influenza virus replication, while knockdown of STING blunts MDA5-mediated IFN-β induction [172]. Duck STING mRNA is present in most tissues and highly transcribed in the stomach, intestine, bursa, liver, and pancreas of 5-day old ducklings, and the blood and pancreas of 2-month-old ducks [173]. Recombinant duck STING localizes to mitochondria and endoplasmic reticulum in DEFs, and induces production of IFN-β and ISGs like Mx and OASL. Knockdown of duck STING blunts IFN-β response to poly(I:C) (a viral RNA mimic) in DEFs. Thus, both duck and chicken STING induce type I interferon directly and potentiate RLR signaling, through MDA5 in chickens, and presumably through RIG-I and MDA5 in ducks.

## 7. Influenza Virus Pattern Recognition—Duck Toll-Like Receptors

### 7.1. TLR7 and TLR 8

Toll-like receptors 7 and 8 (TLR7 and TLR8) are endosomal pattern recognition receptors that recognize single stranded viral RNA [174]. Human TLR7 is highly expressed in the endosomes of plasmacytoid dendritic cells [88], which produce the bulk of IFN-α during influenza infection [175,176,177]. TLR7 and TLR8 in dendritic cells can respond to viral RNA from ruptured cells or infected cells taken up into endosomes [78], but may also function directly in epithelial cells, potentially responding to leaked RNA in the endosome from any defective virions [178]. The TLR8 gene is disrupted in ducks, chickens, and other birds, so only TLR7 is functional [131,178,179]. Pekin duck TLR7 shares 85% amino acid identity with chicken TLR7 and 66% with human TLR7, and is most highly expressed in the lymphoid tissues of the spleen, bursa, and the lung [178]. In contrast, constitutive expression of chicken TLR7 is low in the lungs [180]. Duck splenocytes respond to human TLR7 agonists by increasing IL-1β, IL-6, and IFN-α mRNA. Chicken splenocytes, similarly stimulated, upregulated IL-1β mRNA, but not IFN-α [179]. TLR7-mediated interferon production in chickens may be genotype specific, since heterophils taken from two commercial lines showed different abilities to respond to TLR7 agonist [181], and the TLR7 ligand loxoribine inhibited influenza virus replication in the chicken macrophage cell line HD-11 and in chicken embryos [182]. Chickens upregulated TLR7 in the lungs more strongly than ducks at 16 h post-infection with H7N1 [41]. Populations of TLR7-expressing cells and IFN-α-expressing cells can be detected by in-situ hybridization in the duck intestine, and these are activated or recruited by TLR7 agonists, but not to a significant extent by LPAI A/turkey/Italy/977/1999 (H7N1) [104]. In summary, the TLR8 gene is disrupted in both ducks and chickens, and TLR7 has antiviral functions in both species. Ducks, but not chickens, have high constitutive expression of TLR7 in their lungs, which may contribute to rapid antiviral responses.

### 7.2. TLR3 and TRIF

TLR3 detects double-stranded RNA in endosomes and elicits an interferon response. However, its role in influenza infections is yet unclear. In vitro, human TLR3 transcription is upregulated in A549 and primary alveolar epithelial cells infected with A/Puerto Rico/8/1934 (H1N1), and TLR3 silencing reduces type I and III interferon production [183]. However, in mice infected with A/Scotland/20/74 (H3N2), upregulation of TLR3 expression in lungs at 3 and 4 dpi was correlated with increased inflammation, and TLR3-/- mice had a survival advantage [184]. Chicken TLR3 responds to poly(I:C) in vitro and was upregulated in lung and brain tissue at 24 hpi with VN1203 [185], and in lungs at 24 hpi with A/chicken/Italy/1067/99 (H7N1) [41].

Muscovy duck TLR3 was cloned and characterized in 2012 [186]. It shares 62% amino acid identity with human TLR3, and 87.3% identity with chicken TLR3. The majority of the differences between the duck and chicken TLR3 sequences lie in the cytoplasmic signaling domain, the Toll/Interleukin-1 receptor homology domain (TIR) [187]. Constitutive expression of TLR3 was found in all Muscovy duck tissues except skeletal muscle, by qPCR [186]. Muscovy ducks infected with A/Duck/Guangdong/212/2004 (H5N1) upregulated TLR3 expression in their brains, but downregulated it in lungs and spleens, over three days post infection [186]. Wei et al. [66] also infected Muscovy ducks with A/Duck/Guangdong/212/2004 (H5N1) and A/Duck/Guangdong/383/2008 (H5N1) viruses, and found that TLR3 was upregulated in brain tissues over three days post-infection, but downregulated in lung tissues. A somewhat similar pattern of high upregulation in brains and weak upregulation in lungs is seen in influenza-infected pigeons [188]. Hayashi et al. infected pigeons with two H5N1 viruses, A/Pigeon/Thailand/VSMU-7-NPT/2004 and A/Tree sparrow/Ratchaburi/VSMU-16-RBR/2005, and examined gene expression at 2 dpi, 5 dpi, and 9 dpi. They found that in the pigeon lungs, TLR3 expression was inversely correlated with viral titre. Thus, ducks and pigeons seem to downregulate TLR3 in their lungs upon influenza infection, while chickens upregulate it.

TIR domain-containing adaptor molecule 1 (TICAM1 or TRIF) is an adaptor to TLR3 that mediates NF-κB and IRF activation and apoptosis [189]. Duck TRIF is expressed in most tissues, more highly in liver and spleen [190]. TRIF mRNA is upregulated in duck embryonic fibroblasts stimulated with poly(I:C) or infected with AIV. Overexpression of duck TRIF induces IFN-β expression, which is impaired by point mutation of alanine 517. The A517 mutant TRIF cannot interact with TLR3 through the TIR domain, similar to mutation of residue P434 in the human TRIF homolog [189].

## 8. Other Pattern Recognition Receptors Relevant to Influenza Infection

### 8.1. LSm14A—A Possible Viral RNA Sensor in Ducks

LSm14A is the RNA-binding component of processing bodies that sequester non-translating mRNA, but can also act as a detector of viral RNA and induce IFN-β production in humans [191]. Duck LSm14A is broadly expressed in tissues, and localizes to puncta (presumably P-bodies) in DEFs [192]. Overexpression of duck LSm14A induces IFN-β and NF-κB, and knockdown reduces poly(I:C) sensitivity.

### 8.2. NLRP3

The NOD-like receptor family, pyrin domain-containing 3 (NLRP3) inflammasome is a multi-protein complex that responds to danger and damage signals by activating the cysteine protease caspase-1 [193,194]. This leads to the maturation and secretion of pro-inflammatory cytokines IL-1β and IL-18, and to the production of reactive oxygen species (ROS). There is accumulating evidence that the NLRP3 inflammasome is activated by RIG-I signaling and makes important contributions to the innate immune responses to influenza virus in mice [195,196] and in humans [197] that promote healing. On the other hand, there is also evidence that NLRP3 activation can contribute to a cytokine storm [64]. To our knowledge, no investigation of duck NLRP3 has yet been published. Chinese yellow chicken NLRP3 was cloned in 2015 and found to be significantly diverged from mammalian homologues (54–55% amino acid sequence identity with human and mouse NLRP3) [198]. Constitutive expression analysis by qPCR showed the highest expression levels in the lungs and tracheae of the chickens, and immunohistochemistry confirmed high expression in the ciliated epithelium and basal cells of the trachea and in the alveolar epithelium.

## 9. Interferon-Stimulated Genes (ISGs)

The outcome of the early induction of IFN-β in infected tissues is the establishment of an anti-viral state in the adjacent cells through the production of many interferon-stimulated genes (ISGs). Functional ISG repertoires are crucial for protection from lethal influenza infection in animal models. RNA-seq and quantitative PCR studies have shown that in response to HPAI infections ducks upregulate many important ISGs, like Mx, ISG12-2, IFIT5, OASL [14,38,148]. Table 1 shows the eleven most upregulated genes in our transcriptome analysis of lung tissues from ducks infected with highly-pathogenic A/duck/Hubei/49/2005 (H5N1) [199]. Some of these ISGs have been experimentally characterized in ducks and chickens. Here, we summarize what is currently known about their expression and interactions in the context of influenza virus infection.

### 9.1. Viperin

Viperin (RSAD2) is a broadly antiviral ISG in humans [200,201]. It has enzymatic activity and prevents newly-synthesized influenza virion release, presumably by perturbing the chemistry of lipid rafts, which are required for virus assembly [202]. Mallard duck Viperin shares 75.8% amino acid sequence identity with the human homologue, is inducible by poly(I:C) in vitro, and is upregulated in duck lungs, spleens, intestines, and blood in response to Newcastle disease virus infection [203]. Transcriptome analyses of ducks infected with HPAI H5N1 have identified Viperin as one of the most highly upregulated genes in the lungs [40,199]. Chicken Viperin is 90% identical to the duck orthologue at the amino acid level [204]. It is inducible by poly(I:C), several TLR ligands, and IFN-α in chicken splenocytes, and is induced in chicken lungs and spleens by infection with A/Muscovy duck/Vietnam/453/2004 (H5N1), at 2 dpi. Thus, an inducible and potentially functional Viperin protein exists in both chickens and ducks, and it will be interesting to see whether they can restrict viral replication.

### 9.2. IFIT5

Interferon-induced protein with tetratricopeptide (IFIT) repeats family proteins can bind and sequester single-stranded 5′ triphosphate RNA species lacking 2′-O-methylation at the 5′ end, a viral signature [205,206,207]. Birds possess a single IFIT gene, IFIT5 [131]. Duck IFIT5 is highly upregulated in duck lungs and spleens in response to HPAI H5N1 viruses [37,69]. Recombinant duck IFIT5 inhibited the replication of highly pathogenic A/duck/Hubei/49/2005 and A/goose/Hubei/65/2005 H5N1 viruses in DF-1 chicken cells, comparably to recombinant mouse Mx1 [208]. It also exhibited antiviral activity against several other RNA and DNA viruses.

### 9.3. PKR

The double-stranded (ds) RNA-dependent serine/threonine protein kinase PKR is an interferon-induced protein with antiviral, anti-proliferative, and pro-apoptotic functions [209,210,211]. It is composed of three domains—two N-terminal dsRNA-binding domains (dsRBD) and one C-terminal kinase domain. PKR becomes activated by binding foreign dsRNA in the cytoplasm. The two dsRBDs are joined by a flexible hinge and wrap around a short stretch of dsRNA; two or more PKR proteins binding the same dsRNA molecule lead to mutual phosphorylation and activation of the protein kinase domains [212]. Activated PKR blocks viral replication and cell proliferation by broadly inhibiting protein translation. It does this by phosphorylating the alpha subunit of eukaryotic initiation factor 2 (EIF2α), which shuts down cellular protein synthesis and that of a wide variety of DNA and RNA viruses, including influenza A viruses [209,210]. PKR-knockout mice are highly susceptible to influenza A virus infection [213]. Activated PKR also induces apoptosis through NF-κB-dependent caspase activation. HeLa cells transfected with a trans-dominant negative mutant of PKR exhibited less influenza-infection-induced cell death [214].

PKR is highly upregulated in the lungs of HPAI-infected Pekin ducks, and weakly in the lungs of LPAI-infected Pekin ducks [130]. However, mallard PKR appears to be missing the second dsRBD [130,215]. Since it has not yet been functionally characterized, the consequence of this deletion is currently an open question. We do know, from structural and biochemical studies, that individual dsRBDs are capable of binding dsRNA independently [209,212]. A related PKR protein, from a goose (*Anser anser domesticus*), has been cloned and characterized [215]. Although goose PKR contains both N-terminal dsRBD domains, like mammalian and chicken PKR, it is more closely phylogenetically related to mallard PKR. Goose PKR mRNA was broadly distributed in all tissues of *Anser cygnoides* geese, highly expressed in blood, spleen, lung, bursa of Fabricius and jejunum, and demonstrated antiviral activity against Newcastle disease virus in vitro.

Chicken PKR shares approximately 63% and 62% amino acid identity with mallard duck and goose PKR respectively. A comparison of PKR proteins from seven Japanese chicken breeds found only three amino acid substitution positions, all outside of the dsRBDs and the kinase domain [216]. All but one of the PKR proteins demonstrated antiviral activity against vesicular stomatitis virus in vitro. The exception was PKR from one Koshamo chicken that contained an R507Q substitution at the far C-terminus of the protein. PKR proteins from two other Koshamo chickens that did not contain this substitution retained their antiviral activity, and the mechanism underlying this is still unclear.

In summary, mallard duck PKR is upregulated in IAV-infected tissues, but it is missing one dsRNA-binding domain and remains to be functionally characterized. Goose and chicken PKR proteins have demonstrated antiviral activities against RNA viruses, but influenza A viruses have not yet been tested.

### 9.4. CCL19

Mammalian chemokine CCL19, together with CCL21, act through the CCR7 receptor to orchestrate the trafficking of naïve lymphocytes and dendritic cells to secondary lymphoid organs (reviewed by Förster et al. [217]). While this chemokine axis is generally considered homeostatic, continuously operating to ensure tolerance, it is greatly increased during infection. In ducks, CCL19 and CCL21 were highly induced in H5N1 infected lung tissues [218], which was also confirmed by transcripome analysis [199]. CCL19 expression was similarly induced in Muscovy, mallard or Pekin ducks at 2 dpi [51]. Chickens also highly upregulate CCL19 in response to infection with avian influenza [219]. In mice lacking lymph nodes, pulmonary expression of several chemokines including CCL19 are involved in recruitment of lymphocytes and dendritic cells to establish tertiary lymphoid tissues, which are essential to influenza survival [220]. The consequence of CCL19 expression and recruitment of leukocytes to the infected lung tissues of birds that naturally lack lymph nodes, whether beneficial or harmful, remains an open question.

### 9.5. Mx

Interferon-induced GTP-binding protein Mx is a well-studied and crucial ISG in influenza virus defense in mammals [86,221,222,223,224,225]. Haller et al. [226] provide an excellent review of the antiviral functions of Mx. Mx proteins are thought to be expressed by all vertebrate cell types, and are expressed in all mouse tissues stimulated with poly(I:C) or influenza virus [227,228]. Humans and mice both have two Mx genes encoding two Mx proteins—MxA and MxB in humans, which correspond phylogenetically to Mx1 and Mx2 in mice [229]. Many mechanisms, potentially cooperative, have been proposed for influenza inhibition. In humans, MxA proteins interact directly with influenza nucleoproteins (NP) in the cytoplasm and may block nuclear localization sequences on them [230,231], while nuclear MxA blocks viral RNA transcription, potentially through interactions with the viral NP and polymerase basic subunit 2 (PB2) [232]. A different report shows that primary viral transcription is inhibited in Mx1 (murine) stably-transfected cells, while a post-transcriptional step is inhibited in MxA (human) stably-transfected cells [233].

In ducks that have a strong IFN-β response on the first day post-infection with HPAI H5N1 viruses, Mx1 mRNA is highly upregulated 1–3 dpi, in lung, spleen and brain tissues [40,69,99]. The first investigation of two duck Mx1 alleles found that they did not confer resistance to influenza infection in stably-transfected mouse and chicken cell lines, but the intracellular distribution of the recombinant protein differed from the positive control mouse Mx1 [234]. Single amino acid polymorphisms in Mx1 that confer or abolish antiviral activity have been reported [235,236], so the genetic diversity of duck Mx1 could be relevant for influenza virus ecology. Dillon and Runstadler investigated the diversity of Mx1 among five wild duck species in Alaska by sequencing exon 13 and the 3′ UTR [237]. They found a total of 61 haplotypes across mallards, northern shovelers, northern pintails, American wigeon, and American green-winged teals, with 4 shared among all five species. There was evidence of diversifying selection in exon 13 of Mx in mallard, but no evidence of selection in any of the other species. The authors also did not find any consistent statistical association between Mx haplotype and influenza infection status, although their data did suggest that the strength of the association varies from year to year. Unfortunately, the exon 13 portion that was amplified and sequenced in this paper is homologous to the human MxA protein from residue 585 onward, whereas the variable loop that is responsible for binding to influenza nucleoprotein was recently localized to amino acids 533–578 [238]. Furthermore, nucleoprotein mutations that overcome human MxA inhibition have been described [239,240,241], so the importance and function of duck Mx1 diversity remains an open question.

Chickens have a single polymorphic Mx gene with variable, but mostly weak anti-influenza activity that depends on chicken breed [235,242,243,244,245]. Schusser and colleagues showed that chicken Mx lacks GTPase activity [245]. It was previously shown that mutations disrupting GTP binding and GTPase activity also disrupt the antiviral activity of Mx, and it was assumed that GTP hydrolysis was necessary for antiviral function [228]. A more recent structural analysis found that MxA oligomerization is critical for viral nucleoprotein sequestration, and that GTP binding, but not necessarily hydrolysis, stabilizes MxA multimers [246]. In fact, MxA oligomerization reduced the rate of GTP hydrolysis, and antiviral activity was preserved when MxA bound a synthetic GTP analog that could not be hydrolyzed.

### 9.6. OASL

In humans, two related ISGs called OAS and OASL restrict influenza virus by two different mechanisms. Interferon-inducible 2′-5′-oligoadenylate synthase (OAS) senses dsRNA and synthesizes oligoadenylates that turn on RNaseL, which degrades all mRNA in the cell and globally blocks translation [247,248]. OAS-like protein (OASL) in humans lacks 2′-5′-oligoadenylate synthase activity, but utilizes its ubiquitin-like domains to inhibit RNA virus replication independently of RNaseL, by stabilizing the RIG–I:MAVS interaction in place of, or in co-ordination with, K63-linked polyubiquitin chains furnished by TRIM25 [249,250,251,252]. Thus, OAS1 activates the RNase-L pathway, and OASL activates the RIG-I pathway, when bound to viral RNA.

In ducks and chickens, OASL is the only protein of this family, and it possesses both oligoadenylate synthase activity and the ability to restrict flavivirus replication in an RNaseL independent manner [253,254]. Duck embryonic fibroblasts produce OASL in response to poly(I:C) [255], and it is upregulated in the brain, lung, and spleen tissues of ducks infected with HPAI H5N1 viruses [37,99]. Rong and colleagues recently demonstrated that duck OASL can potentially activate both RNase-L and RIG-I pathways [252]. Wild-type duck OASL restricted the replication of a variety of RNA viruses in vitro, including influenza virus, preferentially by synthesizing 2′-5′-oligoadenylates and activating RNAse-L. When three aspartic acid residues in the N-terminal OAS-like domain were mutated, duck OASL lost its enzymatic activity, but revealed its ability to amplify RIG-I signaling. Both wild-type and mutant duck OASL proteins co-precipitate with RIG-I, but the authors found that WT OASL preferentially activates RNase-L. The triple aspartic acid mutation, which makes duck OASL more similar to non-enzymatic human OASL, reveals its ability to potentiate RIG-I signaling, in an example of functional redundancy under the control of a molecular switch. It is unclear whether wild-type duck OASL also preferentially activates RNase-L in vivo, or if it also activates the RIG-I pathway under certain conditions.

### 9.7. IFITM3

Interferon-inducible transmembrane protein 3 (IFITM3) localizes to endosomes and blocks IAV entry into human cells [256], and limits severity of LPAI pathology in mice [257,258]. Duck IFITM3 shows evidence of positive selection at certain amino acids in regions implicated in influenza virus restriction [40,259]. Amino acids F106 and A112 in the transmembrane (CD225) domain appear to be under persistent positive selection across many bird species, and residue V30 in the N-terminal domain, also important for influenza virus restriction, was invariable in all mallard duck sequences. Blyth and colleagues showed that IFITMs are induced in duck lungs by HPAIV infection, and that duck IFITM3 restricts influenza viruses in vitro [260]. Recombinant duck IFITM3 localized to LAMP1-labelled endosomes in DF-1 cells and reduced replication of H6N2, H11N9, and H1N1 viruses by 50–60%. Endosomal localization was not disrupted by point mutations to four potential YXXθ signal sequences in the N-terminal domain of IFITM3. The other duck IFITMs tested, IFITM1, IFITM2, and IFITM5, did not restrict influenza virus.

Chicken IFITM3 has also demonstrated anti-influenza activity in vitro [261]. A549 cells expressing chicken IFITM3 resisted infection by several luciferase-expressing pseudotyped influenza viruses (H1, H5, H7, and H10). Chicken DF-1 fibroblasts overexpressing IFITM3 resisted infection by A/WSN/1933 (H1N1) compared to controls, while IFITM3 silencing in DF-1 cells rendered them more susceptible to the same infection.

In a side-by-side comparison of chickens and mallard ducks infected with HPAI A/Vietnam/1203/2004 (H5N1), there was a 2-fold decrease of IFITM3 mRNA in chicken ileum, and no change in chicken lungs, whereas in ducks IFITM3 was upregulated 12-fold in lungs and 10-fold in ilea on the first day post-infection [40]. The authors refer to this gene as IFITM1, however the nomenclature for IFITM1 and IFITM3 in both ducks and chickens in this study is reversed compared to the current phylogenetic consensus [260,261]. Thus, both ducks and chickens possess functional IFITM3 proteins that block influenza virus entry into cells, but ducks seem able to induce it earlier in infection, possibly thanks to RIG-I signaling.

## 10. Viral Inhibition of Type I IFN Signaling—Influenza A Virus NS1

Influenza A virus non-structural protein 1 (NS1) is a virulence factor that opposes host cell innate immune signaling and effector functions in multiple ways, as reviewed by Ayllon and Garcia-Sastre [262]. NS1 temporally regulates viral RNA synthesis in host cells [263], controls host protein expression [264], but its most important function is inhibition of type I interferon signaling [262,265]. This is demonstrated by the fact that recombinant influenza A viruses lacking NS1 replicate normally in IFN-deficient cells and animals, but cannot replicate in normal, IFN-competent organisms [103,266,267,268]. Some of the key interactions of NS1 occur with RLR signaling pathway components and with ISGs. NS1 proteins from mammalian and avian strains all target human TRIM25 to block RIG-I ubiquitination [155,269,270]. In mice, NS1 proteins do not interact with TRIM25, but target Riplet instead [155]. Another important function of NS1 in humans is the inhibition of PKR through direct interaction [263].

### 10.1. NS1 in Ducks

NS1 protein function is important for influenza viral fitness in duck cells. A recombinant A/turkey/Italy/977/1999 (H7N1) with a severely truncated NS1 was able to replicate in IFN-deficient Vero cells, but not in duck embryonic fibroblasts [103]. Of specific functions in duck cells, so far we know only that NS1 from strain A/Duck/Guangdong/212/2004 (H5N1) blocks Muscovy duck MDA5 signaling [136]. It is not known whether this is achieved by blocking the ubiquitination of MDA5 CARD domains, or in another manner. It will be interesting to discover whether avian NS1 proteins target the duck RIG-I pathway through TRIM25, and the interferon response more broadly through other proteins, like Lsm14A [271] and PKR [263].

### 10.2. NS1 in Chickens

NS1 protein function is also important for viral fitness in chicken cells. Influenza A viruses with deleted [267] or severely truncated [272] NS1 proteins induce interferon and do not replicate in chicken cells and live chickens. Li et al. [273] described a single-amino-acid mutation—A149V—in the NS1 of HPAI A/Goose/Guangdong/1/1996 (H5N1) that produced a similar severely attenuated phenotype accompanied by high interferon induction and complete lack of disease in chickens. To understand this, we analyzed the available IAV NS1 crystal structures and determined that alanine 149, which is highly conserved across IAV NS1 sequences, is completely buried in the hydrophobic core of the NS1 effector domain. We suggest that a valine substitution may destabilize the protein structure and lead to inactivity or degradation. Zhu et al. [268] described a five-amino-acid internal mutation that destabilized the structure of NS1 and led to its rapid degradation in chicken cells, which likewise severely attenuated the virus and heightened the interferon response.

Rajsbaum et al. [155] demonstrated a species-specificity of NS1 function in chicken cells in vitro. NS1 from avian influenza A/Hong Kong/156/1997 (H5N1) bound chicken TRIM25 strongly and reduced the interferon-β response in a chicken cell line. In the same experiment, NS1 proteins from several mammalian strains interacted only weakly with chicken TRIM25 and did not inhibit interferon effectively. Even though we do not yet know the mechanism of interferon induction by chicken TRIM25, it is clear that other pattern recognition receptors partially compensate for the lack of RIG-I in chickens and that NS1 is essential for viral fitness in this host.

### 10.3. Species-Specificity of the C-Terminal PDZ-Binding Motif

The amino acid sequence and length of NS1 proteins is variable at the C-terminal end. Large-scale sequencing studies have revealed a PDZ-binding motif (PBM) in the last four amino acids of a majority of avian influenza A virus NS1 proteins [274,275]. PDZ domains are protein–protein interaction domains found on host proteins involved in maintenance of cell polarity, tight junctions, and regulation of apoptosis [276,277]. The majority of circulating avian influenza A viruses have NS1 proteins with a C-terminal consensus sequence ESxV [274,275]. The majority of circulating human strains in the past decades share the C-terminal consensus sequence RSxV. Some NS1 proteins are truncated at the C-terminus and have no PBM, like the NS1 of highly-pathogenic A/Vietnam/1203/2004 (H5N1).

The human RSxV motif has a lower affinity than ESxV for PDZ domains [275]. The introduction of ‘ESEV’ sequence into the swine strain A/WSN/1933 (H1N1) increased virulence in mice in an interferon-independent manner [278]. Removing the ‘ESEV’ motif from the NS1 of LPAI A/Turkey/Italy/977/1999 (H7N1) by truncation slightly increased the histopathology in infected chicken lungs without increased replication, and no pathology or replication differences were seen in ducks [279]. Interestingly, replacing ‘ESEV’ with the human ‘RSKV’ in A/Turkey/Italy/977/1999 conferred a replication advantage to the mutant virus in duck fibroblasts, but it induced a higher upregulation of Mx in infected Pekin duck tissues and was shed to a lesser extent than the wild-type virus [280,104]. The addition of either the avian ‘ESEV’ or the human ‘RSKV’ motifs to the NS1 of the highly-pathogenic A/Vietnam/1203/2004 (H5N1) did not affect its virulence or replication efficiency in mice and chickens [281].

In summary, the inhibition of type I interferon signaling by NS1 proteins is clearly an essential attribute for the fitness of mammalian and avian influenza A viruses across hosts. Yet, apart from the viral surface antigens hemagglutinin and neuraminidase, NS1 is the most variable protein of influenza A viruses [274] and has many apparently species-specific functions. The evidence of species-specific and strain-specific differences in IAV NS1 makes its interactions in mallard ducks and chickens a very interesting open question.

## 11. Other Potential Mechanisms of Influenza Disease Resistance in Mallards

### 11.1. Rapid Apoptotic Response

Rapid apoptotic response has been proposed as a potential duck resistance mechanism to influenza proliferation [282]. Ducks infected with HPAI H7N1 had quicker apoptotic and CD8^+^ T-cell responses co-localized to viral antigen in the lungs, by 8hpi, compared to chickens [72]. ISG12-2 is an interferon-stimulated gene that destabilizes mitochondrial membranes in human cells to produce a pro-apoptotic effect [283]. The function of duck ISG12-2 has not been investigated yet, but it is induced in HPAIV infected duck lungs [38]. Ueda et al. report high levels of apoptosis in DEFs infected with A/crow/Kyoto/53/04(H5N1) and A/chicken/Egypt/CL6/07(H5N1), but less apoptosis from LPAI H5N2 and H5N3 viruses [284].

### 11.2. Limiting Inflammation with USP18

Qian et al. cloned and characterized the duck ubiquitin-specific protease 18 (USP18) and proposed that it is involved in controlling inflammatory responses in ducks [255]. In humans USP18 maintains homeostasis by removing ISG15 labels from proteins [285,286]. However, USP18 can also oppose pro-inflammatory NF-κB signaling by cleaving K63-linked ubiquitin chains [287]. Qian et al. found that duck USP18 is broadly expressed in duck tissues, particularly abundant in lung, spleen, and kidney, and that it is inducible by the viral RNA analog poly(I:C) [255]. Overexpression of recombinant duck USP18 suppressed pro-inflammatory cytokine secretion in duck embryonic fibroblasts (DEFs). In vitro experiments with recombinant chicken USP18 showed that it enhanced LPAIV, but not HPAIV, replication [288]. It would be interesting to know if duck USP18 can negatively regulate RIG-I signaling by removing the K63-linked ubiquitin chains furnished by TRIM25. When Qian et al. overexpressed duck USP18 in DEFs they found that it inhibited IFN-β production along with pro-inflammatory cytokines, but that the cells still induced ISGs in response to poly(I:C) [255].

### 11.3. Humoral Adaptive Immunity

Duck antibody structure, repertoire and responses to influenza were previously reviewed [289,290]. In ducks, influenza infection typically occurs in the intestine, and seroconversion does not always occur [47]. However, ducks can respond to influenza vaccines [50], and show some evidence of protection from re-infection by the same strain [5,6] and some hetero-subtypic protection [291,292,293]. Duck antibody responses appear to prevent re-infection with the same strain, and this may drive the diversity of influenza viral subtypes in the wild [294]. Duck antibodies include IgM, IgA and both a full-length and truncated IgY [290]. Chickens do not make the truncated serum IgY antibody. Potentially the truncated duck antibody can neutralize influenza virus, but this has never been tested. In a comparison of duck and chicken antibody responses to LPAI H5N3 infection, chickens had a 10-fold higher response [291]. Similarly, the chicken antibody response to influenza vaccines is stronger and requires less antigen compared to ducks [52]. The cause of the poorer antibody response to influenza in ducks is unknown, but it may be due to the truncated IgY antibody, which would be expected to be impaired in all effector functions.

### 11.4. Cellular Adaptive Immunity

CD8^+^ T cells seem to be retained longer in ducks than in chickens at sites of HPAI infection, suggesting their role in successful viral clearance [72]. In that respect it is noteworthy that CCL19 and CCL21, chemoattractant for CD8^+^ and CD4^+^ T cells [217,295], are upregulated in H5N1-infected duck lungs [218]. Ducks have an extensive repertoire of MHC class I alleles in the wild [296], in comparison to the suspected limited MHC class I diversity in domestic poultry. However, like chickens, ducks predominantly express one of the five genes at the MHC class I locus, per haplotype [296,297]. This theoretically limits the peptide presentation potential of each individual duck. In our survey of MHC alleles, we identified a number of nearly identical alleles that appear in wild and domestic mallards. These alleles possess a unique amino acid motif with cysteines at 95 and 112 in the peptide binding cleft [296]. The crystal structure of one of these alleles showed that the two cysteines form an unusual disulphide bond at the bottom of the cleft, and that the cleft is promiscuous, potentially permitting the binding of a wide variety of influenza-derived peptides [298]. We can speculate that the population-level peptide presentation repertoire of the natural host contributes an important selective pressure on avian influenza viruses.

### 11.5. MicroRNA Regulation

Non-coding RNA species are being recognized as important mediators in multilayered gene-regulatory networks that lead to morphological diversity [299]. The micro RNA repertoire of ducks and chickens is highly conserved, but the expression patterns in tissues in response to HPAI H5N1 infection diverges significantly between these animals, revealing a whole new facet of host–pathogen interaction to be studied [300].

## 12. Final Note on Comparing Duck and Chicken Innate Immune Responses to Influenza Infection

We recognize that a variety of differences between studies preclude direct comparison. Differences between animal species and ages, between viral strains, and important differences in methodologies and reagents make it difficult to compare any two studies side by side. Even within a single study, ducks and chickens have to be inoculated with very different infectious doses of HPAI viruses because of the chickens’ susceptibility to disease, and this complicates direct comparisons.

Host–virus interactions are not limited to immunity, but include protein translation, shuttling of viral components between different cellular compartments, and virion assembly, all of which may have intrinsic efficiency differences between chickens and ducks due to evolutionary divergence of their cellular machinery. We do not attempt to address these variables in our comparison of duck and chicken immune responses to influenza virus. With these limitations in mind, we may consider all of the data about duck and chicken responses to influenza in aggregate to discern some conserved trends and to draw several careful conclusions.

The striking difference in susceptibility to HPAI virus disease between mallard ducks and chickens is primarily due to the lack of RIG-I in chickens. Mallard ducks control viral replication and pro-inflammatory signaling in many HPAI virus infections that would be fatal to chickens, and tolerate significantly higher infectious doses of HPAI viruses in general. We propose that the initial type I interferon signal at the site(s) of infection slows down the first rounds of viral replication and induces a more balanced innate immune response. The demonstrated IFN-α secretion in chickens within 24 h of HPAI virus infection probably relies largely on plasmacytoid dentritic cells, and does not appear sufficient to oppose hyper-inflammation. It is likely that by the time the virus reaches and infects circulating plasmacytoid dendritic cells in chickens, or by the time they otherwise become activated, many other tissues have been infected and hosted at least one round of uncontrolled viral replication, the damage from which precipitates an exaggerated pro-inflammatory response.

Mallard ducks are more resistant than other anseriforme species to HPAI virus disease. The reasons for this are less obvious and likely have to do with more refined evolutionary adaptations which will become clearer as new information emerges.

## 13. Conclusions

The overall picture that emerges is that mallard ducks are very permissive to LPAI replication in their intestines without cost to fitness, and have robust immune responses that give ducks some ability to cope with the spontaneous tendency of LPAI viruses to periodically acquire high pathogenicity and broad tissue tropism. Mallard ducks are not universally resistant to disease caused by all strains of HPAIV, but are much more resistant than chickens, which lack the RIG-I receptor, and relatively more resistant than other bird species. Tight control of pro-inflammatory signaling contributes to the survival of mallards, and may be helped by a lack of viral endothelial tissue tropism and the ducks’ ability to tolerate some extent of central nervous system infection. RIG-I receptor-mediated IFN-β signaling in infected cells and the rapid induction of ISGs is critical to limiting HPAI virus spread and viremia early, and to limiting inflammation. The potential strain-dependent role of viral NS1 in blocking duck RIG-I signaling is an intriguing open question. The duck ISGs IFITM3 and IFIT5 demonstrably function to restrict influenza virus and it will be interesting to see more information emerge about the rest of the duck ISG repertoire, particularly the diverse haplotypes of Mx1. The host–pathogen interactions of mallard ducks and influenza viruses are multifactorial and complex, and the relative resistance to highly-pathogenic strains likely results from a long history of host adaptation through evolutionary fine-tuning.

## Figures and Tables

**Table 1:** Most highly upregulated transcripts in three ducks infected with HPAI A/duck/Hubei/49/2005 (H5N1) at 1 dpi, relative to mock-infected controls, in descending order. Transcriptome data from the NCBI Sequence Read Archive (control transcriptome accession: SRR797835, infected 1 dpi transcriptome accession: SRR797836) were internally normalized to a housekeeping gene, hydroxymethylbilane synthase (ENSAPLT00000008800), before comparison.

Gene Name	Ensembl ID
*RSAD2* (Viperin)	ENSAPLT00000006241
*IFIT5*	ENSAPLT00000001602
*CCL4*	ENSAPLT00000010196
*EIF2AK2* (PKR)	ENSAPLT00000010258
*ISG15*	ENSAPLT00000016054
*IL4I1*	ENSAPLT00000016724
*USP18*	ENSAPLT00000007808
*EPSTI1*	ENSAPLT00000005561
*CMPK2*	ENSAPLT00000006212
*CCL19*	ENSAPLT00000005578
*MX*	ENSAPLT00000016707
